# Identification of long-term survival-associated gene in breast cancer

**DOI:** 10.18632/aging.103807

**Published:** 2020-10-20

**Authors:** Shipeng Ning, Hui Li, Kun Qiao, Qin Wang, Meiying Shen, Yujuan Kang, Yanling Yin, Jiena Liu, Lei Liu, Siyu Hou, Jianyu Wang, Shouping Xu, Da Pang

**Affiliations:** 1 Harbin Medical University, Harbin 150081, China; 2 Department of Breast Surgery, Harbin Medical University Cancer Hospital, Harbin 150081, China; 3 Heilongjiang Academy of Medical Sciences, Harbin 150086, China

**Keywords:** breast cancer, 5-year survival time, complex, enrichment, UGCG

## Abstract

Breast cancer patients at the same stage may show different clinical prognoses or different therapeutic effects of systemic therapy. Differentially expressed genes of breast cancer were identified from GSE42568. Through survival, receiver operating characteristic (ROC) curve, random forest, GSVA and a Cox regression model analyses, genes were identified that could be associated with survival time in breast cancer. The molecular mechanism was identified by enrichment, GSEA, methylation and SNV analyses. Then, the expression of a key gene was verified by the TCGA dataset and RT-qPCR, Western blot, and immunohistochemistry. We identified 784 genes related to the 5-year overall survival time of breast cancer. Through ROC curve and random forest analysis, 10 prognostic genes were screened. These were integrated into a complex by GSVA, and high expression of the complex significantly promoted the recurrence-free survival of patients. In addition, key genes were related to immune and metabolic-related functions. Importantly, we identified methylation of MEX3A and TBC1D 9 and mutations events. Finally, the expression of UGCG was verified by the TCGA dataset and by experimental methods in our own samples. These results indicate that 10 genes may be potential biomarkers and therapeutic targets for long-term survival in breast cancer, especially UGCG.

## INTRODUCTION

Breast cancer (BC) is still the leading cause of cancer-related death in women [1, 2]. In 2008, approximately 1.38 million new cases of breast cancer were confirmed in developing countries, accounting for almost 50% of cancer patients and 60% of mortality [3]. There are large differences in the survival rates of breast cancer throughout the world. It is estimated that the 5-year survival rate in developed countries is 80%, and that of developing countries is less than 40% [4]. Yang et al. pointed out that in 2005, there were 168,013 new cases of breast cancer in China [5]. According to data of the China Central Cancer Registry (NCCR), 4,292,000 newly diagnosed invasive breast cancer cases were reported in 2015 [6]. Although the continuous progress of modern medical technology has improved the survival rate of cancer patients, many of the cured breast cancer patients will eventually relapse and develop drug resistance [7, 8].

The diagnostic methods of breast cancer include ultrasound imaging, nuclear medicine, oestrogen and progesterone receptors, breast biopsy and biomarkers [9]. However, these diagnostic methods cannot accurately predict the survival rate of breast cancer patients. At present, surgical resection is still the classic treatment for breast cancer. Despite the fact that patients with the same molecular subtype of breast cancer receive the same treatment, they can have different results [10, 11]. Many factors lead to the low survival rate of breast cancer patients. On the one hand, breast cancer is heterogeneous [12, 13]. On the other hand, the comorbidity of breast cancer (such as obesity, hypertension, hyperlipidaemia and diabetes mellitus) will affect patients' disease-free survival (DFS) and ultimately affect overall survival (OS) [14]. Therefore, better prognostic factors are needed in order to evaluate therapeutic effects and provide guidance for individualized treatment.

It is worth noting that, although the overall survival rate for breast cancer has improved, the difference between individual survival times of patients is still apparent [15]. In the clinic, no recurrence or metastasis 5 years after treatment means that the risk of cancer patients is greatly reduced. Therefore, there is an urgent need for stratification and biomarker screening in this population. Fortunately, with the development of high-throughput sequencing technology, increasing attention is being paid to the prognosis of breast cancer and the potential molecular indicators for survival. Most importantly, better mechanisms to explore and understand these factors can lead to new therapeutic targets, with the aim of alleviating symptoms and prolonging survival.

In this study, we utilized sequencing data of breast cancer patients from the TCGA and GEO databases. The expression of mRNA, methylation and mutation information in breast cancer patients with survival times longer or shorter than 5 years were analysed by bioinformatics and experiments. Then, the data were screened to identify genes related to the overall survival rate of breast cancer and the possible regulatory mechanisms. Exploring the internal differences between long-term breast cancer and short-term breast cancer may be helpful for revealing predictors of effective survival time and potential therapeutic targets.

## RESULTS

### Differentially expressed genes of high and low survival time in breast cancer patients

**Figure 1:**
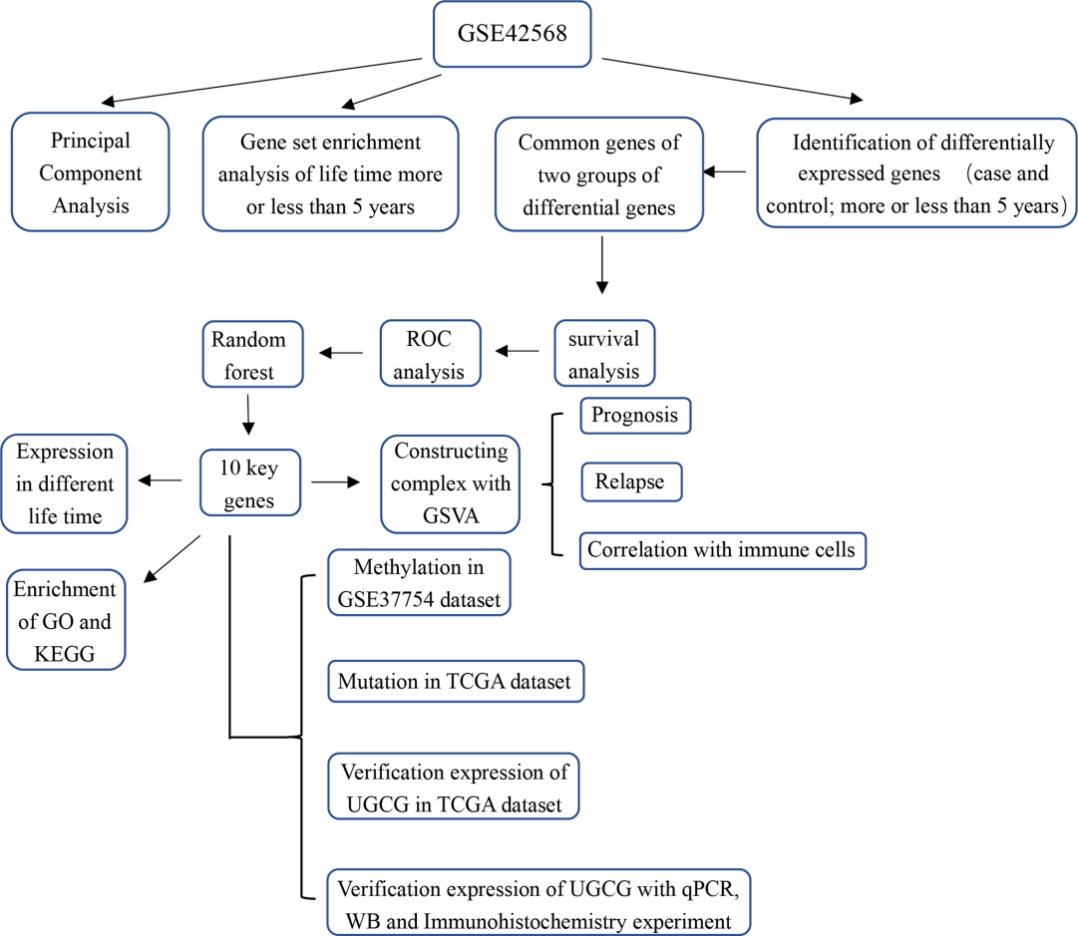
**Study flowchart.**

**Figure 2:**
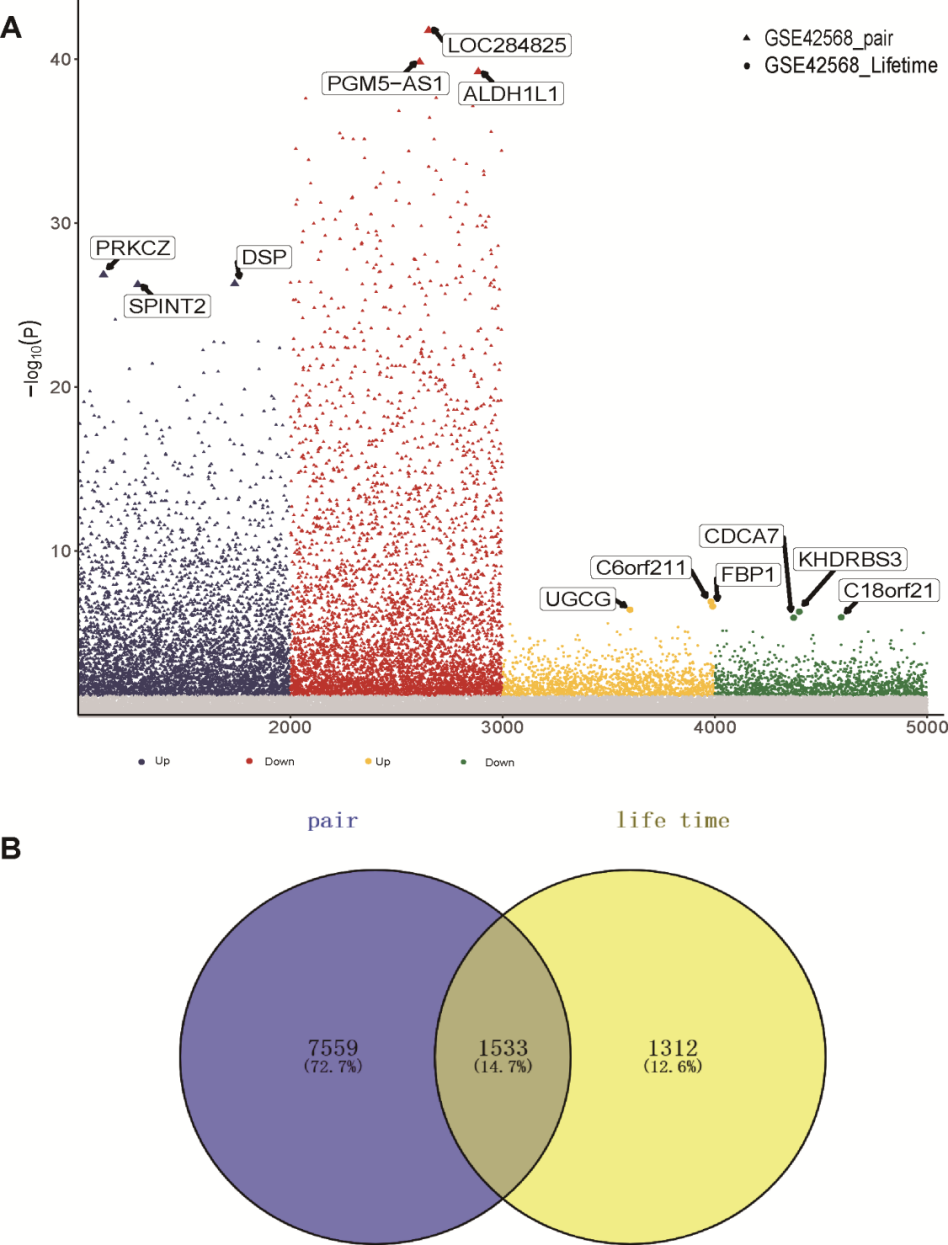
**Gene expression in breast cancer patients.** (**A**) Differentially expressed genes between breast cancer patients and the control group as well as breast cancer patients with high and low survival times. (**B**) The common differentially expressed genes in the two groups.

The study flowchart is presented in Figure 1. To find genes related to the survival time of breast cancer patients, we first performed PCA on the whole sample of GSE42568 in the GEO database. We found that tumour samples and non-tumour samples were independent of each other, indicating that in-depth study of disease samples will be unaffected by the control samples (Supplementary Figure 1A). We further analysed samples of breast cancer patients with a survival time of longer than 5 years and shorter than 5 years, and there were also differences between the samples (Supplementary Figure 1B). Then, we analysed the differentially expressed genes (DEGs) between breast cancer patients and controls (pair) and obtained 9093 DEGs (Supplementary Table 1, Figure 2A). These genes may be related to the occurrence and development of breast cancer. More importantly, we compared the gene expression between patients with a survival time greater than 5 years and patients with a survival time less than 5 years (lifetime). Overall, 2845 DEGs (Supplementary Table 2, Figure 2A) were identified. These genes may be related to the longer survival time of breast cancer. Therefore, we screened 1533 common genes of the two groups of differentially expressed genes (Figure 2B), which may be related to breast cancer and can be used to judge the survival time of breast cancer patients.

### Identification of key genes significantly related to overall survival of breast cancer

**Figure 3:**
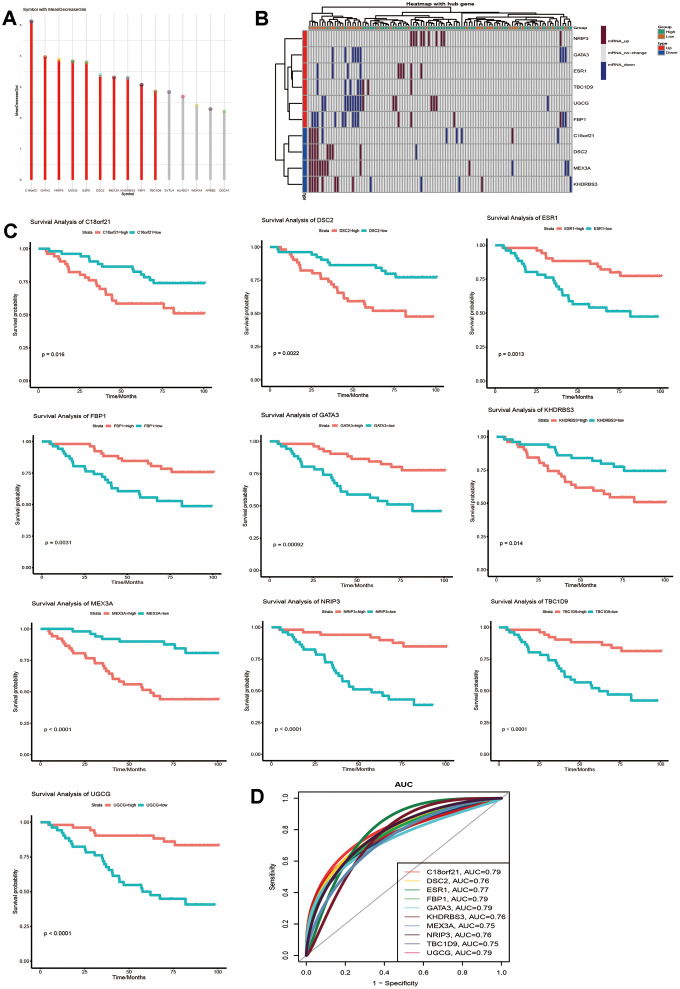
**Identification of key genes that can predict the 5-year survival time of breast cancer patients.** (**A**) Random forest screening for the top 10 genes with a high Gini coefficient of average decline. (**B**) Expression of the 10 selected genes in samples with a survival time of more than or less than 5 years. (**C**) Kaplan-Meier analysis of overall survival for the signatures associated with expression of the 10 genes in breast cancer. (**D**) AUC of the 10 selected genes that affect the survival time of breast cancer.

To further identify whether common genes affect the survival time of breast cancer patients, we identified the relationship between mRNA level and the clinical prognosis of breast cancer. The results showed that 784 common genes were significantly related to the overall survival of breast cancer patients through survival analysis (Supplementary Table 3). In addition, to identify the specificity and sensitivity of these genes to prognosis, we calculated their Area Under Curve (AUC). The top 15 genes of the AUC were selected as candidate genes. Furthermore, 10 genes with high Gini coefficients were screened out through random forest analysis, and these genes are highly representative (Figure 3A). Notably, we also observed the expression of these genes in samples with long or short survival periods (Figure 3B). Nrip3j was highly expressed in patients with a survival time of more than 5 years, while GATA3 was low in patients with a survival time of less than 5 years. The high and low expression of the 10 genes was significantly related to the long survival time of breast cancer (Figure 3C). The AUC value of C18orf 21, FBP 1, GATA 3 and UGCG was the highest, at 0.79 (Figure 3D). Thus, these genes have potential diagnostic value and may become biomarkers for breast cancer. However, further validation is required in future studies.

### The effect of all 10 genes on the overall survival of breast cancer

**Figure 4:**
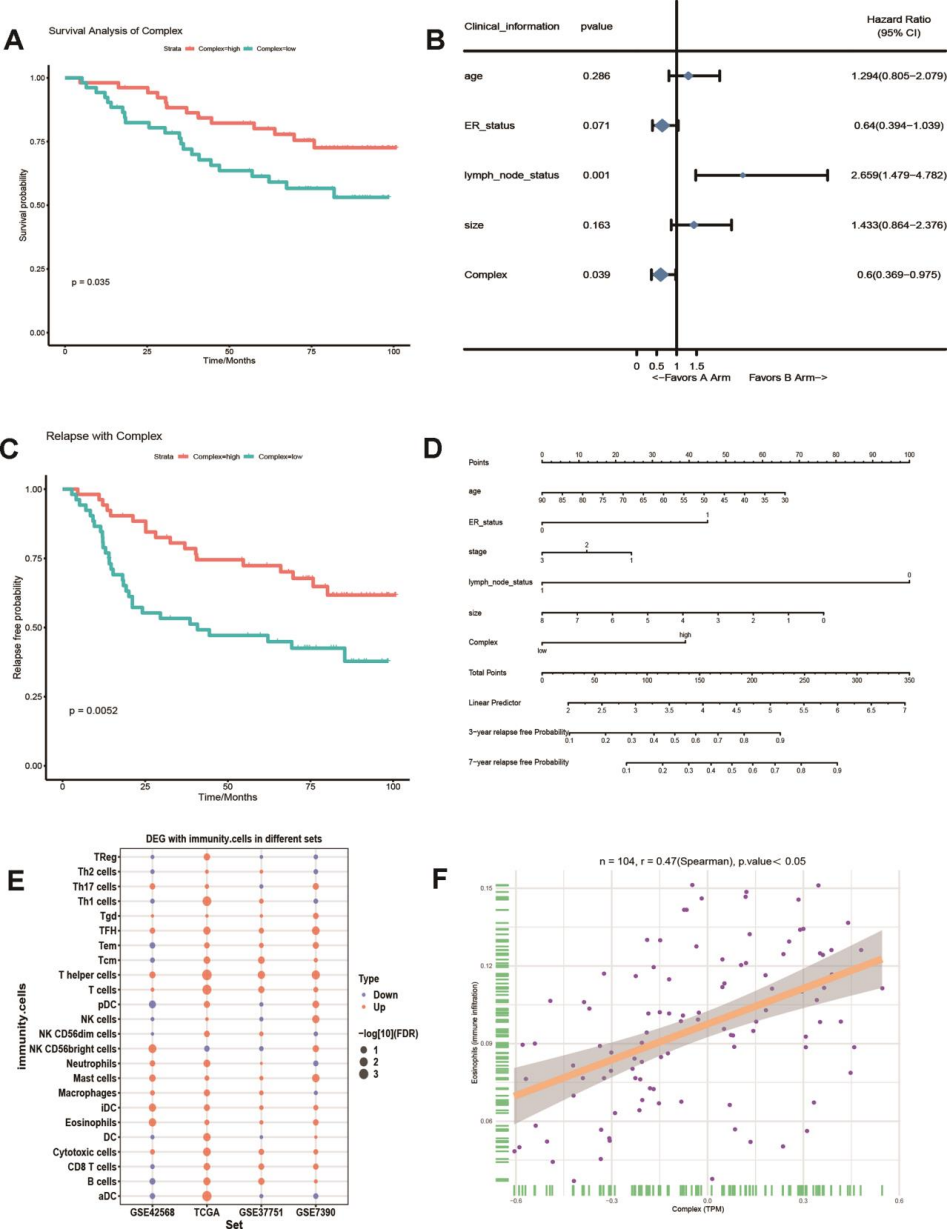
**The effect of 10 genes on the prognosis of breast cancer.** (**A**) GSVA integrates 10 genes into a complex, which affects the overall survival rate of breast cancer patients. (**B**) The risk ratio of the gene complex and clinical information to breast cancer prognosis. (**C**) Kaplan-Meier relapse analysis of the effect of the complex on breast cancer relapse. (**D**) A Cox regression model was used to analyse the effect of multiple variables on breast cancer relapse. (**E**) The difference of 24 kinds of immune cells in breast cancer with longer than 5 years and shorter than 5 years survival time. (**F**) The correlation curve between the complex and eosinophilia.

To evaluate the role of all 10 genes in the prognosis of breast cancer, we combined 10 genes into a complex through GSVA and found that the complex significantly affected the overall survival of breast cancer patients (Figure 4A). Single factor Cox regression analysis showed that lymph node status was a strong risk factor for breast cancer prognosis (Figure 4B). Kaplan-Meier relapse analysis for breast cancer patients according to complex expression levels showed that the complex influenced relapse-free breast cancer (Figure 4C). We used a Cox regression model for multivariate relapse analysis and a Cox regression coefficient to generate a nomogram (Figure 4D). In multivariate relapse analysis, age, ER status, stage, lymph node status, size and complex expression levels were considered as risk factors for relapse. The nomogram predicted the relapse probability of breast cancer patients in 3 and 7 years. The results showed that the low level of complex expression was closely related to the relapse rate of breast cancer patients. In addition, the difference of immune cells in different survival time of breast cancer was calculated (Figure 4E). A variety of immune cells were up-regulated in four groups of data. In the correlation analysis with immune cells, we found that the complex had the highest positive correlation with eosinophils (Figure 4F). Therefore, these key gene complexes are related to the prognosis of breast cancer as well as the relapse.

### Biological process of key genes in the prognosis of breast cancer

**Figure 5:**
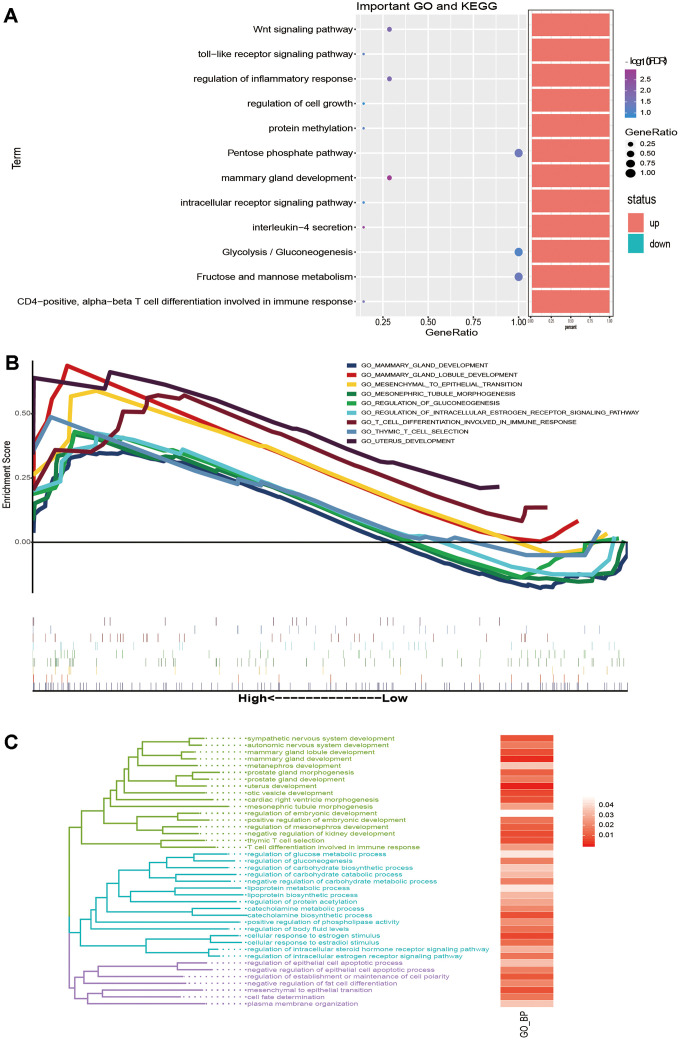
**The biological function and signalling pathway of the key genes affect the prognosis of breast cancer.** (**A**) The bubble chart shows BP and KEGG enriched by key genes. (**B**) GSEA of genes expressed by breast cancer patients with survival times greater than 5 years. (**C**) The same BP as GSEA was clustered into three types of biological functions.

To explore the biological process involved in the influence of key genes on the survival time of breast cancer patients, we analysed the GO and KEGG pathway enrichment of the 10 selected key genes (Supplementary Table 4). The results showed that FBP1 is involved in glycolysis and other carbohydrate metabolism pathways. UGCG, GATA 3, ESR 1 and DSC 2 are involved in many biological processes, including mammary gland development and T cell differentiation involved in the immune response (Figure 5A). Then, GSEA was performed on the genes expressed by breast cancer patients to determine the different biological functional states of breast cancer patients whose survival times are longer or shorter than 5 years. The results showed that the genes of patients with a survival time of longer than 5 years mainly promoted biological functions, such as mammary gland development, glycogenesis and T cell differentiation involved in the immune response (Figure 5B). In addition, biological functions with the same results as GSEA could be clustered into three functional types by similarity, which may play similar roles (Figure 5C). Based on the above results, 10 key genes may affect the prognosis of breast cancer through immune and metabolic pathways.

### Potential regulatory factors affecting the prognosis of key genes

**Figure 6:**
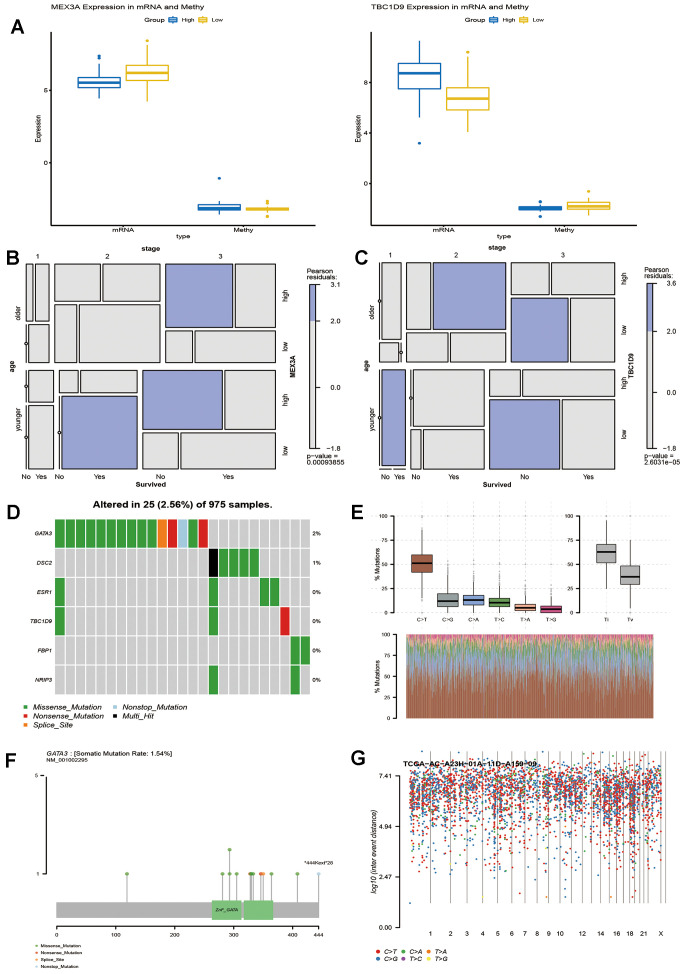
**Potential factors affecting key genes that influence the survival time of breast cancer patients.** (**A**) Methylation level and expression of MEX3A and TBC1D9 in breast cancer patients with survival times greater than or less than 5 years. Mosaic analysis identified the relationship between the expression of MEX3A (**B**) or TBC1D9 (**C**) and the prognosis and clinical information of breast cancer. (**D**). Six key genes were sequenced according to their mutation frequency. Different colours represent different methods of mutation. (**E**) The transition and crosscut graphs show the distribution of SNV in breast cancer with six transition and crosscut events. The stacked bar graph (bottom) shows the mutation spectrum distribution of each sample. (**F**) The Lollipop map shows the mutation distribution and protein domain of GATA3 with a high frequency of mutation. (**G**) The Rainfall map of TCGA-AC-A23H-01A-11D-A159-09 breast cancer sample. Each point is a mutation colour coded according to the SNV classification.

Since we observed that key genes are involved in the prognosis of breast cancer, we used these transcription factors as candidate genes to understand whether they have epigenetic effects. First, we explored the methylation level of key genes (Supplementary Table 5). Analysis of GSE37754 methylation data showed that the methylation levels of MEX3A and TBC1D 9 were negatively correlated with the mRNA expression levels in breast cancer. MEX3A showed a state of high methylation and low transcription, while TBC1D 9 showed a state of low methylation and high transcription (Figure 6A). Thus, MEX3A and TBC1D 9 are affected by methylation in breast cancer survival. In addition, through mosaic analysis, it was found that the high expression of MEX3A was positively correlated. with the mortality of breast cancer patients in advanced stage and elderly patients, and the low expression was beneficial to the prognosis of young patients (Figure 6B). The high expression of TBC1D 9 was beneficial to the survival of early breast cancer patients, and its low expression promoted increased late mortality (Figure 6C). The results of these analyses are consistent with the trend that these two genes predict a long or short life span

In addition, exploring somatic mutations is helpful to understand the occurrence and development of breast cancer. We analysed the mutation frequency of 10 key genes, wherein 6 had different degrees of mutation (Figure 6D). The transition plot classified single nuclear variants (SNV) into six categories (Figure 6E). Among them, the C > T mutation accounted for more than 50% of the total mutations. Among the six mutations, GATA 3 had the highest mutation frequency, and the longest mutation site was *444Kext*28 (Figure 6F). Kataegis is a mutation process observed in cancer, and 55% of breast tumours will lead to high mutations in local genomic regions [16]. The distribution of the mutation spectrum of breast cancer samples can also be identified by a rainfall map (Figure 6G). Regardless of the methylation modification or site mutation of genes, they all play an important role in the survival of breast cancer.

### Verification of the expression profile of key genes

**Figure 7:**
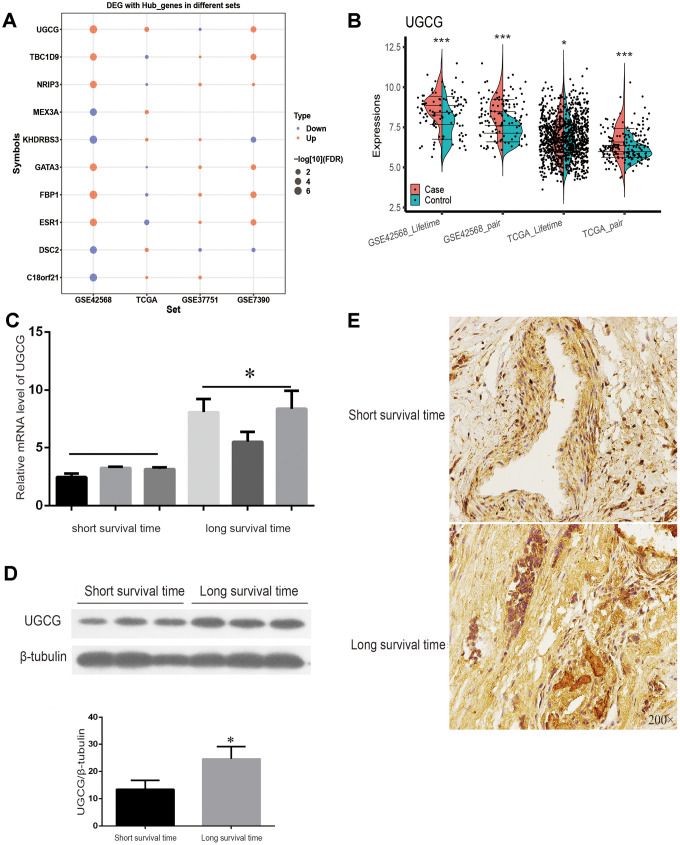
**TCGA sets and experiments to verify the expression of key genes.** (**A**) Expression of 10 key genes in breast cancer patients with a survival time of more than or less than 5 years with four data sets. (**B**) TCGA and GSE7390 were used to verify the significant expression of UGCG. (**C**) The mRNA level of UGCG in breast cancer patients with a survival time greater than or less than 5 years was detected by qRT-PCR. (**D**) Western blot was used to detect the expression of UGCG in breast cancer patients with a survival time greater than or less than 5 years. (**E**) Immunohistochemistry images of UGCG levels in breast cancer tissues with a survival time greater than or less than 5 years. Scale bar = 200 μm. *P < 0.05, ***P < 0.001.

Through the public data of TCGA, GSE37751 and GSE7390, the expression trend of 10 genes in breast cancer patients with long and short survival times were verified. (Figure 7A). Among them, the mRNA level of UGCG was significantly different in the two groups of datasets (P < 0.05) (Figure 7B). The data show that UGCG mRNA expression was significantly correlated with breast cancer disease and positively correlated with breast cancer prognosis. Therefore, UGCG plays a major role in the prediction of survival time of breast cancer. To further verify the explicit correlation between UGCG and long-term survival of breast cancer patients, we carried out qPCR, Western blot and immunohistochemistry experiments. Surprisingly, the experimental results are consistent with the expectations. The transcription of UGCG in breast cancer patients with a survival time of longer than 5 years was significantly higher than that of breast cancer patients with a survival time of shorter than 5 years (Figure 7C). Western blot (Figure 7D) and immunohistochemistry (Figure 7E) also confirmed the expression of UGCG. These data indicate that UGCG may be a biomarker to predict the survival time of breast cancer patients.

## DISCUSSION

In the past few decades, the causes of changes in breast cancer incidence and mortality have been analysed, which has important implications for the application of adjuvant therapy and the judgement of risk factors for breast cancer. Many genes that cause diseases in cancer are expressed simultaneously and form a complex network of actions [17, 18]. Using large-scale sequencing data provides a unique opportunity to unravel the gene prognosis relationship with breast cancer, which can be used to identify the influencing factors of tumour prognosis and finally determine treatment targets. In this study, we found differentially expressed genes in breast cancer patients who had survival times longer or shorter than 5 years. Through a series of analyses, 10 genes related to the longer survival time of breast cancer were identified.

The risk of metastasis and relapse remains high after standard treatment of breast cancer, with more than 30% of breast cancer patients experiencing recurrence [19]. Regression analysis showed that high expression of the 10 genes promoted the survival of breast cancer patients and reduced their recurrence. In addition, the complex showed a positive correlation with eosinophils. Eosinophilic infiltration is considered to be associated with a good prognosis in breast cancer [20]. Surface active protein D (SP-D) induces apoptosis of anaphylactic eosinophils and leukaemic cells through the p53 pathway, which may be related to the poor prognosis of breast cancer [21].

Among these key genes, the UDP-glucose ceramide glycosyltransferase (UGCG) produces glycosyl ceramide (GlcCer), a precursor of all sphingolipids (GSL) [22]. The growth and therapeutic response of human tumours may depend on the expression of GSL [23]. Consistent with the results of previous studies, UGCG was highly expressed in breast cancer in this study [24]. Other studies have shown that UGCG is over expressed in metastatic breast cancer tissues, leading to a poor prognosis [25]. Our results are different. Although UGCG is highly expressed in breast cancer patients, its high expression is related to long survival time in multiple sets of data. In addition, our experimental data also confirmed that UGCG expression was high in breast cancer patients with a long survival time. However, the specific mechanism by which UGCG promotes breast cancer cell proliferation and good prognosis is not clear and warrants further study.

Genes play a regulatory role through different biological functions and signalling pathway networks. Through enrichment analysis, we found that the key genes selected in this study participate in biological functions similar to those of GSEA. There is a close relationship between mammary gland development and breast cancer [26, 27]. T cell differentiation involved in the immune response plays an important role in the occurrence and development of breast cancer, and it is also a key factor affecting the survival time of breast cancer patients [28]. The number of infiltrating adaptive immune cells, mainly composed of T lymphocytes, in breast cancer is lower than that in other tumour types, but infiltration is observed in the majority of breast cancers [29]. T cells seem to change the expression profile of breast cancer cells and promote brain metastasis through the blood-brain barrier [30]. At present, tumour-specific T cells are the ultimate goal of tumour immunotherapy.

On the other hand, breast cancer is a complex and heterogeneous disease. In the past few decades, gene expression, methylation and mutation analyses have led to significant findings, which can increase the accuracy of prognoses of clinically relevant patients [31–33]. In addition to the discovery of gene expression, identifying differences in gene methylation was also a focus of this study. MEX3A and TBC1D 9 were methylated and affected the prognosis of breast cancer. In line with other studies, MEX3A expression increased in breast cancer patients [34]. MEX3A showed low expression in breast cancer patients with long survival, which may be related to methylation modifications. In addition, consistent with our results, the high expression of TBC1D 9 reduced the mortality of breast cancer patients and prolonged their survival time [35]. Although the mechanism of TBC1D 9 in breast cancer is not clear, it is significantly correlated with ESR 1, which is a risk factor for breast cancer [36].

Remarkably, we also found that six genes affected by mutations were associated with longer survival in breast cancer. Among them, the mutation frequency of GATA 3 was the highest. GATA 3 is a driving gene of breast cancer, which was also confirmed in our analysis [37–39]. The expression level of GATA 3 is closely related to oestrogen receptor α (ER α). The lack of GATA 3 expression is related to poor prognosis [40, 41]. Cancer-driven gene mutations are usually divided into functional gain mutations and functional loss mutations [42]. In the study of mutations of breast cancer, mutations of GATA 3 caused the largest number of response genes, perhaps making it a tumour suppressor [43]. Our analysis also suggests that mutations in GATA 3 affect the survival time of breast cancer patients.

Our results highlight genes associated with greater than 5-year survival in breast cancer patients and the regulatory mechanisms involved in these genes. By exploring gene expression, methylation, mutation and biological pathways, it is possible to establish biomarkers related to breast cancer prognosis. However, there are also some defects in our research. Although the expression trend of key genes had been verified in other datasets, the sample size of the main analysis data was small, and the results may be biased. In this regard, follow-up research is necessary to elaborate the specific mechanisms by which the 10 key genes identified here relate to long-term survival of breast cancer patients. Perhaps, we can predict the long or short survival time of breast cancer patients according to the expression of key genes, and we can also find the target drugs of key genes to intervene the survival time.

## CONCLUSION

We identified 10 genes related to the overall survival time of breast cancer. High expression of the 10 gene complex significantly promoted the recurrence-free survival of breast cancer patients. Among them, the expression of UGCG was validated by the TCGA data set and through experiments. In addition, enrichment analysis showed that the key genes were related to immune and metabolism-related functions.

## MATERIALS AND METHODS

### Data source and differential expression analysis

The transcriptome data set of breast cancer was obtained from the genomics data repository Gene Expression Omnibus (GEO) and The Cancer Genome Atlas (TCGA). We used five years as the dividing line to construct the differential expression profiles. The GEO dataset GSE42568 provided gene expression of 104 breast cancers and 17 normal breast biopsies, among them, there were 64 long survival time and 40 short survival time patients. GSE37751 included 16 long survival time and 45 short survival time breast cancers. GSE7390 included 166 long survival time and 32 short survival time breast cancers. There were 1027 breast cancer samples in TCGA, which included 230 long survival time and 797 short survival time breast cancers. The genes expressed in the high and low survival periods and normal samples were analysed by Principal Component Analysis (PCA) using the ggbiplot function of the “ggbiplot” package (V3.5.3). After background correction and quartile data normalization, difference analysis was performed with the lmfit function of the “limma” package (V3.44.1) [44]. The screening threshold was P < 0.05.

### Gene Set Enrichment Analysis (GSEA)

To reveal biological correlations of the obtained gene expression profiles, the microarray data were compared using GSEA (http://software.broadinstitute.org/gsea/). GSEA uses weighted Kolmogorov-Smirnov to test whether the distribution of genes in the gene set is different from the normal distribution [45]. The gene sets significantly enriched for genes associated with greater than 5 years of life [false discovery rate (FDR) < 0.05] were selected as enriched gene sets. ssGSEA was used to quantify the immune infiltration (expression profile with immune cells) and calculate the correlation between the complex and immune cells [46].

### Gene set variation analysis (GSVA) spectrum conversion

The GSVA (V1.25.4) software package for R was used as a non-parametric, unsupervised method for estimating the variation of key gene sets [47]. The input for the GSVA algorithm was a gene expression matrix of log2 microarray expression values and a collection of pre-defined gene sets or databases of pre-existing gene sets (MSig). GSVA scores were calculated non-parametrically using a Kolmogorov-Smirnoff (KS)-like random walk statistic and a negative value for a particular sample and gene set.

### Survival and ROC curve

The “survival” and “survminer” R packages were used to calculate the impact of hub genes on survival. P value less than 0.05 was considered significant. The AUC of the hub gene with long or short survival time samples was estimated using the R package “pROC” (V1.64.0). Taking sensitivity as the ordinate and specificity as the abscissa, the ROC curve of the receiver was established. A single factor Cox model was used to determine whether hub genes and clinical variables were related to the prognosis of breast cancer.

### Methylation and somatic mutation analysis

The Infinium Human Methylation450 BeadChip (Illumina Inc., San Diego, CA) was used to measure DNA methylation in tissue samples. We obtained the methylation data of GSE37754 from the GEO database. We calculated methylation levels from raw data using M-values after performing background correction for each sample using noob method and normalization for colour bias using SWAN method. Based on the limma R package, we retrieved the aberrant methylated genes with the screening P value < 0.05.

We analysed gene mutations of breast cancer expression in TCGA by the R package “maftools” (V2.4.0) [48]. Visualization in Maftools facilitates the generation of publication-quality images with easy to use and customizable functions.

### Gene ontology (GO) and Kyoto Encyclopedia of Genes and Genomes (KEGG) enrichment

The “Clusterprofiler” package (V3.16.0) of R language was used for enrichment analysis of GO functions and KEGG pathways [49]. “Clusterprofiler” is a software package of Bioconductor, which can perform statistical analysis and visualization of functional clustering on gene sets or gene clusters. When the P adj value was less than 0.05, the GO term or KEGG pathway was identified as significantly enriched by these genes.

### Nomogram construction

We conducted multivariate Cox regression analysis to screen the important independent factors of breast cancer recurrence. The positive stepwise selection method of Cox multiple regression model was used to screen all variables. Based on the important independent factors, we built a nomograph [50].

### Patients and samples

This study was approved by the human ethics review committee of Harbin Medical University. Ten cases of breast cancer were collected from the Department of Breast Surgery, Harbin Medical University Cancer Hospital with the informed consent of the patients. These cases included 3 patients with survival time greater than 5 years and 3 patients with survival time of less than 5 years. The standard requirements for patients in the study were: (1) histologically confirmed breast cancer; (2) no history of other malignancies or other serious diseases that may affect the follow-up results. Follow up was conducted every six months, and the follow-up time is defined as the date from pathological diagnosis to death or the last follow-up.

### Ethics statement

The breast cancer tissue samples were collected according to the International Ethical Guidelines for Biomedical Research involving Subjects. All samples were collected with the informed consent of patients. This study was approved by the Cancer Institute of the Harbin Medical University Cancer Hospital (Group) Ethics Committee and was carried out in accordance with the regulations of the Ethics Committee.

### Quantitative real-time PCR with reverse transcription (qRT–PCR)

Total RNA was extracted with a Trizol Kit (TaKaRa, Kyoto, Japan) according to the manufacturer's protocol. In a 10 μl reaction mixture, approximately 1000 ng of RNA was reverse transcribed into cDNA using a 5 × Primescript RT master mixture (TaKaRa, Kyoto, Japan). The expression of select genes was quantitated using the SYBR Premix Ex TaqTM II kit (TaKaRa, Kyoto, Japan). The reaction conditions were as follows: initial denaturation and enzyme activation at 95 °C for 30 s, denaturation at 95 °C for 5 s, annealing at 60 °C for 30 s. Finally, the gene expression was normalized to GAPDH. See Table 1 for primers for real-time PCR.

**Table 1:** Primer sequence of UGCG and GAPDH.

UGCG_F	TTCTTGGTGCTGTGGCTGATGC
UGCG_R	AGAGAGACACCTGGGAGCTTGC
GAPDH_F	AGAAGGCTGGGGCTCATTTG
GAPDH_R	AGGGGCCATCCACAGTCTTC

### Western blot analysis

In the presence of protease inhibitors, cleavage buffer (CST, MA, USA) was used to cleave breast cancer tissue. Then, samples were centrifuged for 15 min (13000 × g, 4 °C). The BCA protein analysis kit (Keygen Biotechnology) was used to determine the protein concentration. The same amount of protein was electrophoresed on an 8-12% SDS-PAGE, transferred to a PVDF membrane, and blocked with 5% skim milk for 1 h at room temperature. Specific antibodies were incubated overnight at 4 °C, and then samples were incubated for 2 h with the appropriate horseradish peroxidase-conjugated secondary antibody (1:3000 diluent). Chemiluminescence was detected by a Tanon 4600 imaging system (Millipore).

The following primary antibodies were purchased: anti-UGCG (Proteintech, Rosemont, USA) and anti-beta tubulin (Proteintech, Rosemont, USA).

### Immunohistochemistry

Paraffin-embedded breast cancer tissue fixed with formalin was cut into 4 μm thick sections for immunohistochemical staining. The sections were dewaxed, rehydrated, incubated with 90% formic acid for 10 min, washed in buffer, blocked with 3% hydrogen peroxide and 10 μg/ml avidin for 30 min, sealed with normal horse serum prepared with 10% normal saline for 30 min, and incubated with anti UGCG antibody (Proteintech, NO.128691-1-AP) overnight for 30 min. The next day, the samples were washed with PBS and incubated with biotinylated secondary antibody for 10 min at room temperature. Then, the sections were stained with diaminobenzidine and 20% haematoxylin. Microscopy was performed with an Olympus Light microscope (Olympus, Center Valley, PA).

### Replicates

Each experiment was performed three times. The results were substantiated by repetition under a range of conditions.

### Statistical analysis

R (v.3.5.1) software was used for statistical analysis. Quantitative data are shown as the means ± standard deviation (SD), and classified data are shown as counts (percentage). A single factor Cox regression model was used to calculate the influence of clinical characteristics and mRNA expression level on the prognosis of breast cancer patients. *P < 0.05 was considered statistically significant.

### Ethics approval

Breast cancer tissue samples were collected according to the International Ethical Guidelines for Biomedical Research involving Subjects. All samples were collected with informed consent of patients. This study was approved by the Cancer Institute of the Harbin Medical University Cancer Hospital (Group) Ethics Committee and was carried out in accordance with the regulations of the Ethics Committee.

## Supplementary Material

Supplementary Figures

Supplementary Table 1

Supplementary Table 2

Supplementary Table 3

Supplementary Table 4

Supplementary Table 5
