# Supplementary material for: Identification of long-term survival-associated gene in breast cancer
Source: Aging (Albany NY). 2020 Oct 20;12(20):20332–49. doi: 10.18632/aging.103807 (PMC7655188; doi:10.18632/aging.103807)
Supplement: Supplementary Figures [file aging-12-103807-s001..pdf]

SUPPLEMENTARY FIGURE

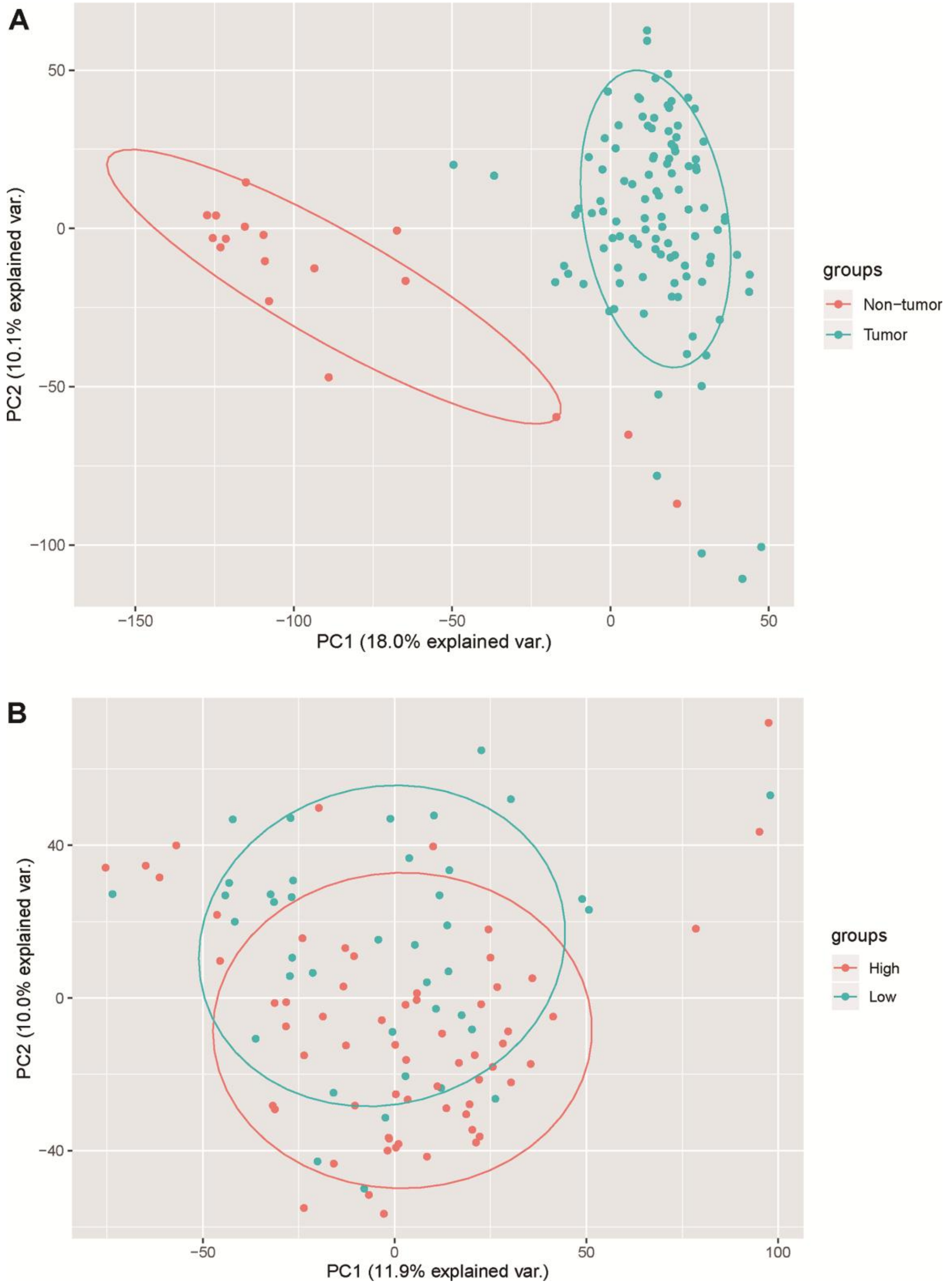

Supplementary Figure 1. PCA analysis of all samples in the GSE42568 data. (**A**) PCA of breast cancer samples and healthy control samples. (**B**) PCA of breast cancer patients with survival time greater than 5 years and less than 5 years.
